# The Relationship Between Metabolic Health and Mortality Among Older Hispanics in the US and Mexico

**DOI:** 10.1093/geroni/igab046.3167

**Published:** 2021-12-17

**Authors:** Maria Carabello

**Affiliations:** University of Michigan, Ann Arbor, Michigan, United States

## Abstract

Studies consistently show that Hispanics, especially first-generation Mexican immigrants, face lower mortality risks in mid-to-late life than US-born non-Hispanic whites. This extended lifespan defies expectations given Hispanics’ disadvantaged socioeconomic status relative to whites and thus is referred to as the Hispanic paradox. However, it remains an open question as to whether the Hispanic paradox in mortality mirrors a lower chronic disease burden. To address this gap, this study will combine and leverage two harmonized longitudinal population-based data sources of late-middle-aged and older adults in the United States and Mexico; the Health and Retirement Study and the Mexican Health and Aging Study. First, I evaluate differences in the association between metabolic syndrome (MetS) and mortality risk for older adults living in Mexico, first-generation Mexican immigrants to the US, US-born Mexican Americans, and US-born whites. Second, I explore the extent to which the proportion of deaths attributable to MetS in each of these groups can be explained by differences in socioeconomic and health/behavioral characteristics. This study uses Cox proportional hazards models to estimate the mortality risks of MetS across groups, as well as the associated population attributable fractions (PAFs) to investigate potential differences within a decomposition framework. Developing this detailed understanding of metabolic health and the associated mortality risks across multiple generations of older Mexican immigrants may help us identify modifiable lifestyle and behavioral factors to better manage these conditions and alleviate possible complications as current and future generations of Mexican immigrants age in the US.

